# Extending our tools and resources in the non-conventional industrial yeast *Xanthophyllomyces dendrorhous* through the application of metabolite profiling methodologies

**DOI:** 10.1007/s11306-017-1313-9

**Published:** 2018-02-12

**Authors:** Eugenio Alcalde, Paul D. Fraser

**Affiliations:** School Biological Sciences, Royal Holloway, University London, Egham Hill, Egham, Surrey TW20OEX UK

**Keywords:** *Xanthophyllomyces dendrorhous*, GC–MS, Carotenoids, Metabolite profiling

## Abstract

**Introduction:**

*Xanthophyllomyces dendrorhous* is a non-conventional industrial yeast. It has the unique ability among yeasts to produce geranylgeranyl pyrophosphate derived terpenoids such as carotenoids and in particular the high value pigment astaxanthin.

**Objective:**

In order to fully exploit the industrial potential of *Xanthophyllomyces* using modern industrial biotechnology approaches the further development of “omic” resources in this organism are required to build on the now sequenced and annotated genome. To contribute to this goal, the present study has developed and implemented an efficient metabolite profiling system comprised of, quenching, extraction and associated GC–MS and UPLC analysis.

**Method:**

Four quenching methods and five extraction methods compatible with GC–MS and UPLC profiling were tested and validated by analysing steady state metabolite changes of *Xanthophyllomyces* cultivated at laboratory scale in liquid shake culture at lag, exponential and early and late stationary phases.

**Results:**

A customised Automated Mass Spectral Deconvolution and Identification System (AMDIS) library has been created for *Xanthophyllomyces*, over 400 compounds are present in the library of which 78 are detected and quantified routinely in polar and non-polar derived extracts. A preliminary biochemical network has been constructed. Over a standardised laboratory growth cycle, changes in metabolite levels have been determined to create reference point for future strain improvement approaches and the initial biochemical network construction. Correlation analysis has illustrated that astaxanthin formation correlates positively with different sectors of intermediary metabolism (e.g. the TCA cycle intermediates and amino acid formation), “short” saturated fatty acids and β-carotene, while other metabolites are reduced in response to astaxanthin production. These sectors of intermediary metabolism offer potential future targets for the manipulation resulting in the generation of strains with improved titres of given terpenoids.

**Discussion:**

In summary a robust metabolite profiling system for *Xanthophyllomyces* is in place to further our understanding and potential exploitation of this underutilised industrial yeast.

**Electronic supplementary material:**

The online version of this article (10.1007/s11306-017-1313-9) contains supplementary material, which is available to authorized users.

## Introduction

*Xanthophyllomyces dendrorhous* is a basidiomycetous yeast first reported over 40 years ago by Herman Jan Phaff and collaborators (Miller et al. 1976; Phaff et al. 1972). *Xanthophyllomyces* and the green alga *Haematococcus pluvialis* are the main natural sources of astaxanthin (Schmidt et al. 2011). Astaxanthin is one of the most important industrial carotenoids presently used, it is known for its reddish pink colour and is mainly used in the aquaculture feed industry. The value of the global carotenoid market in 2014 was estimated at $1.5 billion and is expected to reach nearly $1.8 billion in 2019 (Business Communications Company 2015). Presently, chemical synthesis remains the production method of choice despite the high cost, and unfavourable environmental credentials. However, the growing consumer demand for more natural products (Cataldo et al. 2016) and the dwindling fossil fuel reserves, from which the precursors for chemical synthesis originate, are driving the market to implement new renewable sources (Mannazzu et al. 2015). *Xanthophyllomyces* with its amenability to large scale mutagenesis programmes, rudimentary pathway manipulation, and improvements in culture condition have been developed in order to improve *Xanthophyllomyces* as a valuable industrial production platform (Schmidt et al. 2011; Verdoes et al. 2003).

The genome of *Xanthophyllomyces* has been fully sequenced and recently published (Sharma et al. 2015). The main biosynthetic pathways derived from acetyl-CoA are terpenoids and lipids. The genes involved in ergosterol (the main sterol), (Loto et al. 2012), astaxanthin (Alcaíno et al. 2008; Ojima et al. 2006; Verdoes et al. 1999) and fatty acids have been identified (Sharma et al. 2015). The biosynthetic genes assigned providing valuable tools for the metabolic engineering of the pathway. Carotenoids are forty-carbon (C_40_) terpenoids formed from the C_5_ precursor isopentenyl diphosphate (IPP), making them members of the isoprenoid family of natural products. In *Xanthophyllomyces*, terpenoids are synthetize via the mevalonate pathway (Sharma et al. 2015). The IPP derived prenyl lipids are formed by the action of farnesyl diphosphate synthase (FPPS), the farnesyl diphosphate (FPP) formed which has a C_15_ alkane chain is the precursor for sterols. Further extension of prenyl chain to the C_20_ geranylgeranyl diphosphate (GGPP) which is formed by GGPP synthase (GGPPS) occurs in *Xanthophyllomyces*. Condensation of GGPP catalysed by phytoene synthase results in the formation of phytoene, a colourless C_40_ carotene. Four desaturation and two cyclization catalysed by phytoene desaturase and lycopene cyclase, respectively, form β-carotene. Astaxanthin synthase a P450-type mixed function oxygenase produces two hydroxylations and two ketolations at the 3,3′ and 4,4′ positions on the β-ionone ring, respectively to form astaxanthin. Carotenoids are produced within the organism to provide protection against photooxidative stress and inactivated free radicals via electron transfer (Niyogi et al. 1997). It is the formation of isoprenoids in general and particularly those beyond GGPP that give *Xanthophyllomyces* the unprecedented opportunity to be the platform of choice for the production of high value isoprenoids that can be utilised across multiple industrial sectors.

Metabolomics/metabolite profiling is a key component of the omics toolkit; enabling the large scale determination of steady state levels of metabolites as well as the perturbations in these metabolites resulting from changes in their environment or in response to pathway manipulation (Villas-Bôas et al. 2005). Key attributes of microbial metabolomics systems include the effective arrest (quenching) of metabolism, efficient extraction of metabolites across a broad dynamic range and the analysis/visualisation of the data (Mashego et al. 2007). These metabolite profiling techniques have been developed in industrial/model microorganisms like *Escherichia coli* (Prasad Maharjan and Ferenci 2003; Taymaz-Nikerel et al. 2009; Winder et al. 2008) and *Saccharomyces cerevisiae* (Villas-Bôas et al. 2005). However, *Xanthophyllomyces* has the unique characteristics of being a naturally high terpenoid producer and a “Crabtree negative” industrial yeast (Van Urk et al. 1989). can In the present study a metabolite profiling pipeline for *Xanthophyllomyces* has been established and its utility demonstrated. With these procedures in place biochemical characterisation of strain improvement outputs and associated Genome Scale Metabolic Reconstruction (GSMR) networks can now be performed

## Results

### Analysis of quenching methods

Both primary or intermediary metabolism have an extremely fast turnover (Winder et al. 2008). Efficient quenching methods are essential for accuracy when determining steady state metabolite levels in microorganisms. Previous studies addressing the quenching of metabolism in yeasts (Canelas et al. 2008), have focused on four different quenching methods to evaluated the arrest of metabolism in cultured cells. The comparator (control material) being cellular material harvested by centrifugation without the application of the quenching methods prior to freezing the biomass has been described in the methods section.

**Fig. 1:**
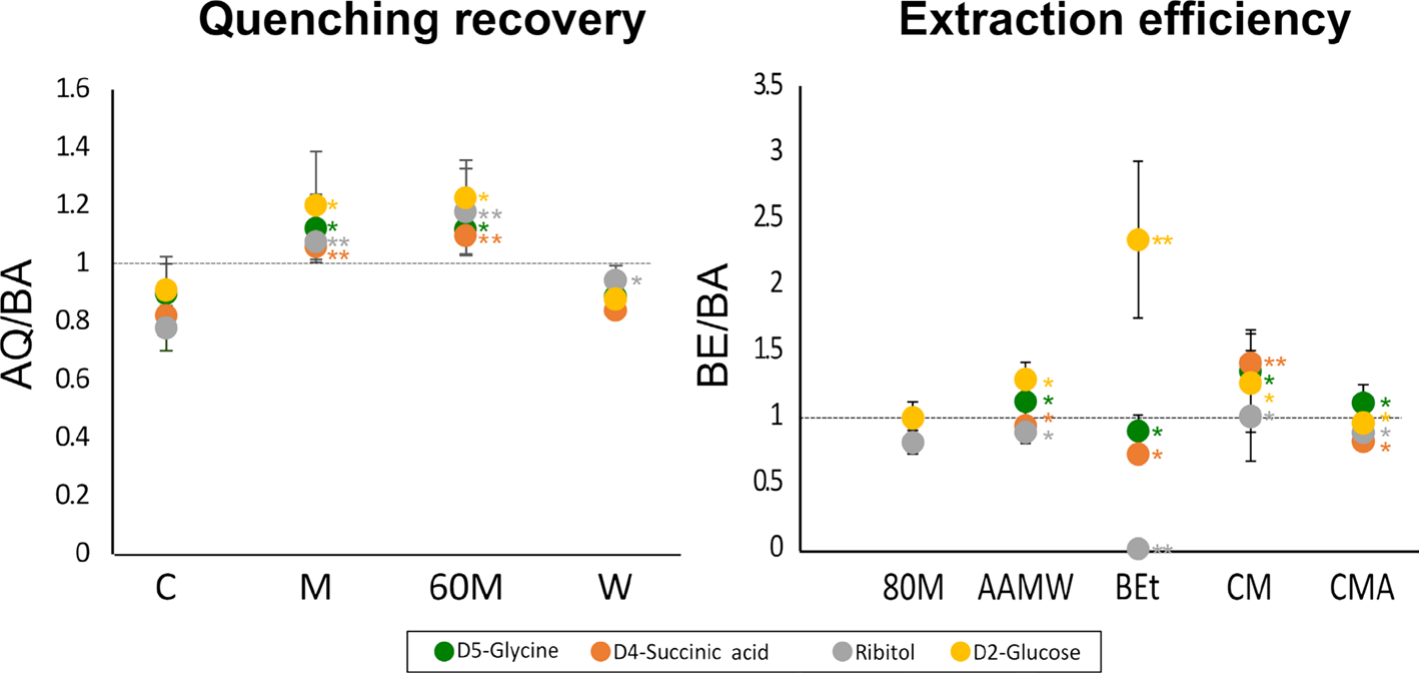
Quenching recovery and extraction efficiency calculated as the ratio of the standards spiked after quenching (AQ) or before extraction (BE) and before analysis (BA) respectively. *C* control, *60M* cold 60% methanol in 0.9% NaCl, *M* cold methanol, *W* washing with 0.9% NaCl, *80M* 80% methanol, *AAMW* acidified acetonitrile:methanol:water, *Bet* Boiling ethanol, *CM* chloroform:methanol, *CMA* chloroform:methanol acidified. *p\0.05; **p\0.01 [C method used as control for recovery and 80M method used as control for efficiency]

**Fig. 2:**
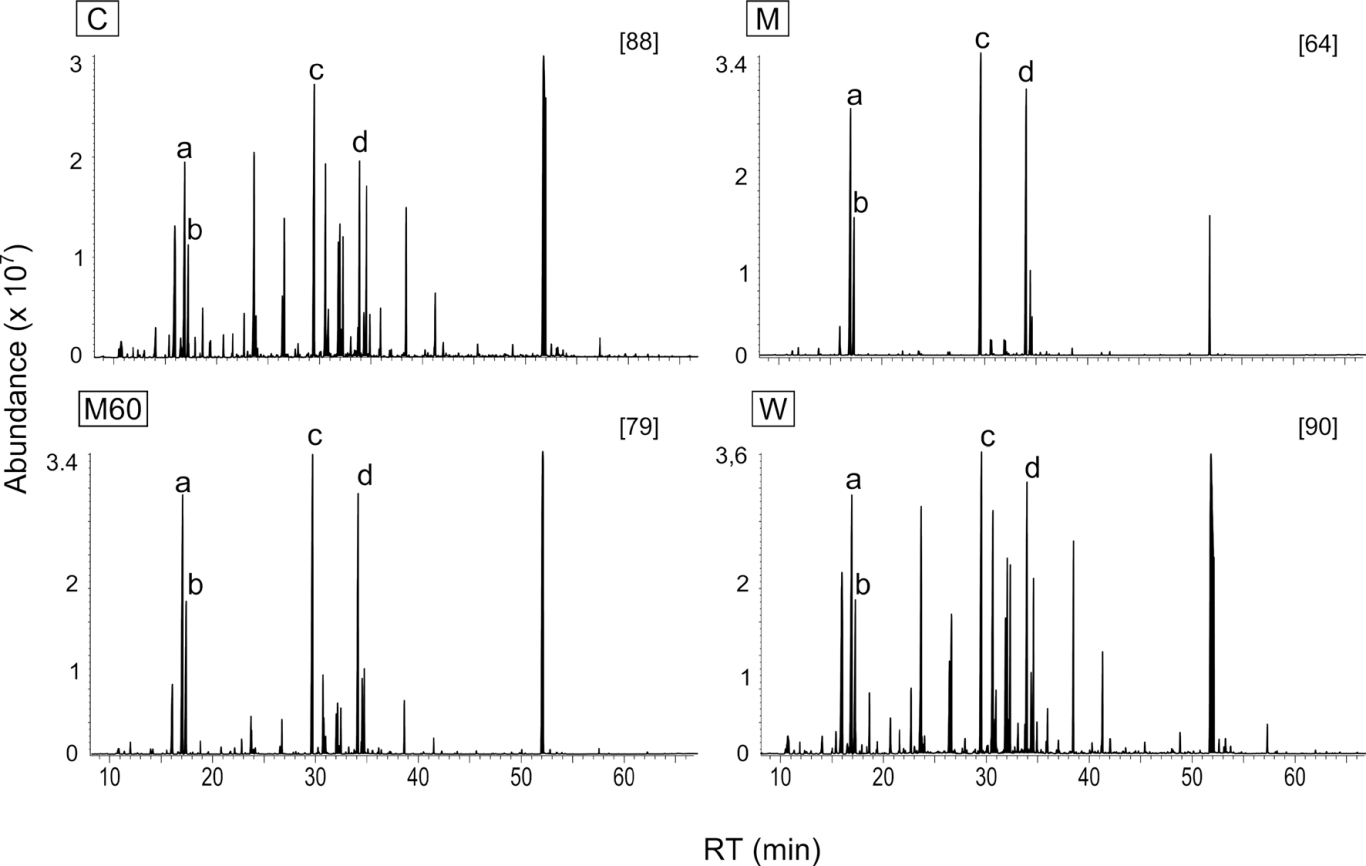
A comparison of the GC–MS profiling following extracts prepared with different quenching methods. In squares: *C* control, *60M* cold 60% methanol in 0.9% NaCl, *M* cold methanol, *W* washing with 0.9% NaCl. In brackets: number of targeted compound in the library. Standards added indicated by letters: **a** d_5_-glycine; **b** d_4_-succinic acid; **c** ribitol; **d** d_2_-glucose

All quenching methods showed a recovery value between 0.77 and 1.22, with the arrest of metabolism in *Xanthophyllomyces*, based on the recovery of the authentic standards used (Fig. 1). The effect of the quenching methods over the general metabolism was also evaluated. The complete overview of the polar extraction of metabolites was provided from the GC–MS analysis, which through library matching showed a high number of identified compounds in samples treated with the wash NaCl solution (W). In this case the initial searches identified over 90 targeted polar compounds (Fig. 2). This quenching method provided the most satisfactory results (in terms of compound numbers) and was logistically appropriate for the study of *Xanthophyllomyces* and its industrial exploitation.

### Extraction optimisation

The 80% methanol (80M), acidified acetonitrile:methanol:water (AAMW), chloroform:methanol (CM) and chloroform:methanol acidified (CMA) methods all showed a good performance. The highest extraction efficiency values for all the standards used were achieved with the CM method (Fig. 1). The boiling ethanol (Bet) method gave the greatest differences in efficiency. However it was the most effective method for d_2_-glucose extraction but the important standard ribitol could not be detected.

**Fig. 3:**
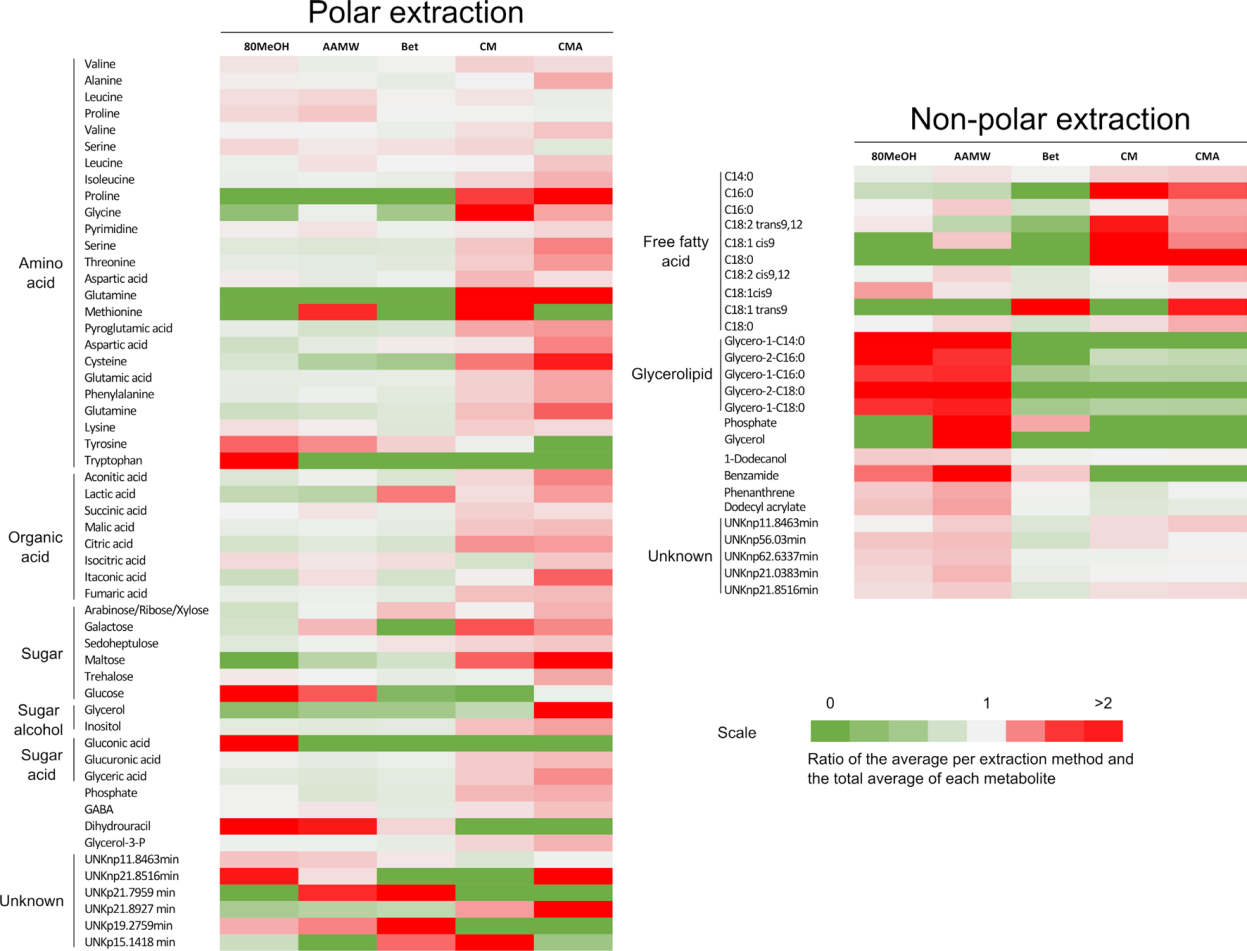
Heat map illustrating the levels of polar and non-polar metabolites obtained with different extraction methods. Values were normalized to the dry weight of samples and pareto-scaled. Values represent means and standard deviations obtained from six replicates

A comparison between all the metabolites extracted by the extraction methods tested showed important differential extraction properties between the compound classes (Fig. 3). The chloroform/methanol-based methods (CM and CMA), were more efficient at extracting polar compounds, such as amino acids, organic acids, sugars and sugar derivatives, and free fatty acids. The highest efficiency for glycerolipids and unknown non-polar compounds was detected using the 80M and AAMW approaches. BEt showed less efficiency for all the classes of metabolites detected/tested.

Base on extraction efficiency, logistically properties and the range of metabolites detected the CM method in combination with the NaCl wash step was the most convenient procedure for extraction in *Xanthophyllomyces*.

### GC–MS library for xanthophyllomyces dendrorhous

The GC–MS library for *Xanthophyllomyces* was created in an AMDIS format, using retention time locking and concurrent chromatography with a hydrocarbon standard mixture, in order to generate of retention indices (Table S1). Identification of chromatographic components was carried out by firstly performing automated matching of mass spectrum to those present in the NIST and/or Golm metabolome databases (http://gmd.mpimp-golm.mpg.de/analysisinput.aspx). These putative matches were then confirmed with authentic standards. Alternatively, authentic standards representing metabolites known to be present in *Xanthophyllomyces* and possessing chemistry amenable to GC–MS analysis, were chromatographed and their characteristic on-line MS spectrum added to the library. The library presently contains more than 400 metabolites and its curation is on-going. This analytical platform allowed the identification and quantification of 76 metabolites (Table S2) among the polar and non-polar extracts of the different ages *Xanthophyllomyces* cultures. In accordance with the recommendations proposed by the metabolomics standards initiative (Bino et al. 2004; Sumner et al. 2007), chromatographic components were annotated, providing valuable metadata and validation of peak identities (Table S1). To provide an indication of the biological and technical variation within the system a series of analyses were carried out on material cultivated under standardised conditions.

Over a 100 GC–MS peaks were detected in the extracts of the quenching samples in *Xanthophyllomyces* strains, 68 were identified as known components and 8 unknown. Amino acids, fatty acids, organic acids and sugars are mainly represented. Furthermore, the terpenoids, β-carotene, phytoene, astaxanthin, ubiquinone (Q9) and ergosterol, were detected by UPLC-PDA. The metabolites identified included a range of intermediates and end products of the central and secondary metabolism (Table 1). This enabled the construction of a quantitative biochemical network for *Xanthophyllomyces* for the display of changes in steady state levels of detectable metabolites present.

**Table 1:** Relative metabolite changes occurring at designated growth stages (exponential and early and late stationary stages) compared with a standardised 1 day (lag stage) culture as control

Metabolite	Fold change			Metabolite	Fold change		
	2 Days	4 Days	7 Days		2 Days	4 Days	7 Days
Amino acids				Phosphate			
Alanine	0.48 ± 0.28	0.69 ± 0.35	0.29 ± 0.17	Phosphate	0.40 ± 0.09	0.61 ± 0.14	0.74 ± 0.27
Aspartic acid	0.70 ± 0.13	1.00 ± 0.19	1.16 ± 0.18	Polyols			
Beta-alanine	1.17 ± 0.48	14.36 ± 4.35	1.19 ± 0.35	1-Dodecanol	1.38 ± 0.36	0.99 ± 0.14	1.02 ± 0.12
Cysteine	0.84 ± 0.12	2.94 ± 0.26	6.57 ± 2.34	Ribitol	0.79 ± 0.11	0.15 ± 3 0.01	A
GABA	1.48 ± 0.28	0.20 ± 0.11	0.20 ± 0.05	Total	0.84 ± 0.08	0.22 ± 0.01	0.09 ± 0.01
Glutamic acid	0.84 ± 0.11	0.76 ± 0.20	0.81 ± 0.14	Phosphorylated compounds			
Glutamine	*	P	P	Glucose-6-phosphate	0.78 ± 0.22	0.25 ± 0.02	0.28 ± 0.12
Glycine	0.48 ± 0.03	1.81 ± 0.16	0.83 ± 0.49	Glycerol-3-phosphate	0.55 ± 9 0.04	0.57 ± 9 0.02	0.49 ± 0.08
Homoserine	A	0.96 ± 0.27	0.54 ± 0.26	Total	0.56 ± 0.04	0.56 ± 0.02	0.48 ± 0.08
Isoleucine	P	P	P	Sugars			
Leucine	0.74 ± 0.06	1.39 ± 1 0.15	1.39 ± 0.20	Arabinose/ribose/xylose	0.61 ± 1 0.08	0.61 ± 1 0.04	0.19 ± 0.11
Lysine	1.18 ± 0.03	0.59 ± 0.09	1.07 ± 0.53	Disaccharide56.509min	0.94 ± 0.15	0.39 ± 0.04	0.29 ± 0.06
Methionine	0.48 ± 0.09	1.67 ± 0.37	0.97 ± 0.45	Disaccharide56.902min	A	A	A
Phenylalanine	0.83 ± 0.12	3.51 ± 0.66	2.00 ± 0.61	Fructose	0.55 ± 0.04	A	A
Proline	0.63 ± 0.09	0.24 ± 0.06	0.59 ± 0.05	Glucose	0.30 ± 0.06	0.07 ± 0.01	0.06 ± 0.01
Putrescine	A	A	A	Maltose	P	*	*
Pyroglutamic acid	0.88 ± 0.14	0.86 ± 0.40	0.87 ± 0.33	Me-fructose	A	A	A
Serine	0.89 ± 0.16	2.39 ± 0.26	2.52 ± 0.78	Me-galactose	P	P	P
Threonine	0.36 ± 0.04	0.37 ± 0.10	0.27 ± 0.12	Sedoheptulose	*	*	P
Valine	0.79 ± 0.26	0.94 ± 0.15	1.32 ± 0.18	Trehalose	0.68 ± 0.17	0.67 ± 0.15	0.75 ± 0.11
Total	0.71 ± 0.09	1.05 ± 0.05	0.88 ± 0.15	Total	0.59 ± 1 0.13	0.52 ± 0.11	0.57 ± 0.08
Fatty acids				Sugar acids			
C14:0	P	P	P	Erythronic acid	A	A	A
C15:0	1.11 ± 0.15	1.84 ± 0.30	1.96 ± 0.19	Glucuronic acid	*	P	P
C16:0	1.37 ± 0.23	0.97 ± 0.05	0.91 ± 0.13	Glyceric acid	1.38 ± 0.31	2.98 ± 0.57	1.63 ± 0.28
C17:0	1.34 ± 0.46	2.81 ± 1.72	2.16 ± 0.82	Total	0.82 ± 0.19	2.99 ± 0.49	2.05 ± 0.28
C18:0	1.27 ± 0.16	0.63 ± 0.02	0.57 ± 0.14	Sugar alcohols			
C18:1 trans9	1.74 ± 0.36	1.43 ± 0.27	1.25 ± 0.26	Glycerol	0.59 ± 0.06	0.00 ± 0.00	0.01 ± 0.00
C18:1cis9	1.41 ± 0.35	0.95 ± 0.07	0.99 ± 0.19	Inositol	0.71 ± 0.01	2.01 ± 0.09	1.61 ± 0.43
C18:2 cis9,12	1.30 ± 0.20	1.12 ± 0.08	1.02 ± 0.12	Total	0.62 ± 1 0.05	0.45 ± 0.02	0.37 ± 0.10
C18:2 trans9,12	2.72 ± 0.72	2.04 ± 1.01	1.69 ± 1.28	Non-amino acid N-containing compounds			
C20:0	1.43 ± 0.19	0.84 ± 0.05	0.70 ± 0.15	Dihydrouracil	*	P	P
C22:0	1.68 ± 0.26	1.28 ± 0.21	1.26 ± 0.39	Pyrimidine	1.24 ± 0.09	2.97 ± 1 0.39	2.55 ± 0.60
C24:0	2.09 ± 0.39	1.53 ± 0.21	1.41 ± 0.33	Total	1.24 ± 0.09	4.14 ± 0.54	3.979 0.73
Total	1.41 ± 0.24	1.11 ± 0.08	1.03 ± 0.16	Terpenoids			
Glycerophospholipids				Astaxanthin	1.63 ± 1 0.28	2.30 ± 0.32	3.32 ± 0.30
Glycero-1-C14:0	0.95 ± 0.15	0.75 ± 0.16	0.60 ± 0.09	Beta-carotene	1.09 ± 3 0.20	1.02 ± 0.22	1.10 ± 0.15
Glycero-1-C15:0	0.95 ± 0.15	0.79 ± 0.07	0.80 ± 0.07	Ergosterol	0.73 ± 3 0.15	0.44 ± 0.06	0.60 ± 0.09
Glycero-1-C18:0	0.93 ± 0.16	0.75 ± 0.06	0.79 ± 0.08	Phytoene	0.49 ± 0.13	0.49 ± 1 0.24	0.12 ± 0.13
Glycero-1-C18:1cis9	0.85 ± 0.28	0.66 ± 0.18	0.45 ± 0.12	Ubiquinone	1.03 ± 1 0.20	0.49 ± 0.10	0.87 ± 0.12
Glycero-2-C16:0	0.84 ± 0.18	0.85 ± 0.11	0.89 ± 9 0.06	Total carotenoids	0.98 ± 0.15	1.03 ± 0.14	1.45 ± 0.12
Glycero-2-C18:0	0.81 ± 0.13	0.87 ± 0.09	0.90 ± 0.07	Total terpenoids	0.76 ± 0.15	0.48 ± 0.07	0.67 ± 0.09
Total	0.94 ± 0.15	0.77 ± 0.07	0.80 ± 0.07	Unknown compounds			
Organic acids				UNKnp11.8463min	1.03 ± 0.20	2.84 ± 2.67	0.31 ± 0.34
Aconitic acid	1.06 ± 0.43	5.17 ± 1.26	7.45 ± 3.10	UNKnp21.0383min	1.13 ± 0.11	2.23 ± 0.97	0.50 ± 0.18
Citric acid	0.84 ± 0.21	2.07 ± 0.31	3.50 ± 1.14	UNKnp56.03min	1.01 ± 0.17	0.88 ± -± 0.06	0.86 ± 0.12
d-Arabinonic acid	A	A	A	UNKnp62.6337min	1.20 ± 0.18	1.13 ± 0.16	0.95 ± 0.13
Fumaric acid	2.15 ± 0.14	3.21 ± 0.55	2.57 ± 0.45	UNKp15.1418min	0.33 ± 0.07	0.13 ± 0.04	2.21 ± 1.38
Itaconic acid	*	P	P	UNKp21.7959min	A	A	0.52 ± 4 0.12
Lactic acid	0.46 ± 0.36	A	A	UNKp21.8927min	*	P	P
Malic acid	1.33 ± 0.05	2.05 ± 0.25	2.00 ± 0.34	UNKp27.5420min	A	1.40 ± 0.45	A
Succinic acid	0.74 ± 0.07	0.60 ± 0.10	0.64 ± 0.08	Total	0.82 ± 0.11	2.42 ± 0.85	1.91 ± 0.25
Total	1.00 ± 0.11	1.78 ± 0.15	2.38 ± 0.54				

Data were compiled from multiple analytical platforms. The ratio data are presented as mean ± SD. Student’s *t* test analysis was carried out. Significant changes are presented in bold (P value < 0.05). P, when a metabolite is present in the sample and not in the control at the concentration used; A, theoretical value when a metabolite is unique to control at the concentration used; *, indicates metabolite no detected in both cultures at the concentration used

### Changes in steady state metabolites during the production of *Xanthophyllomyces* biomass

**Fig. 4:**
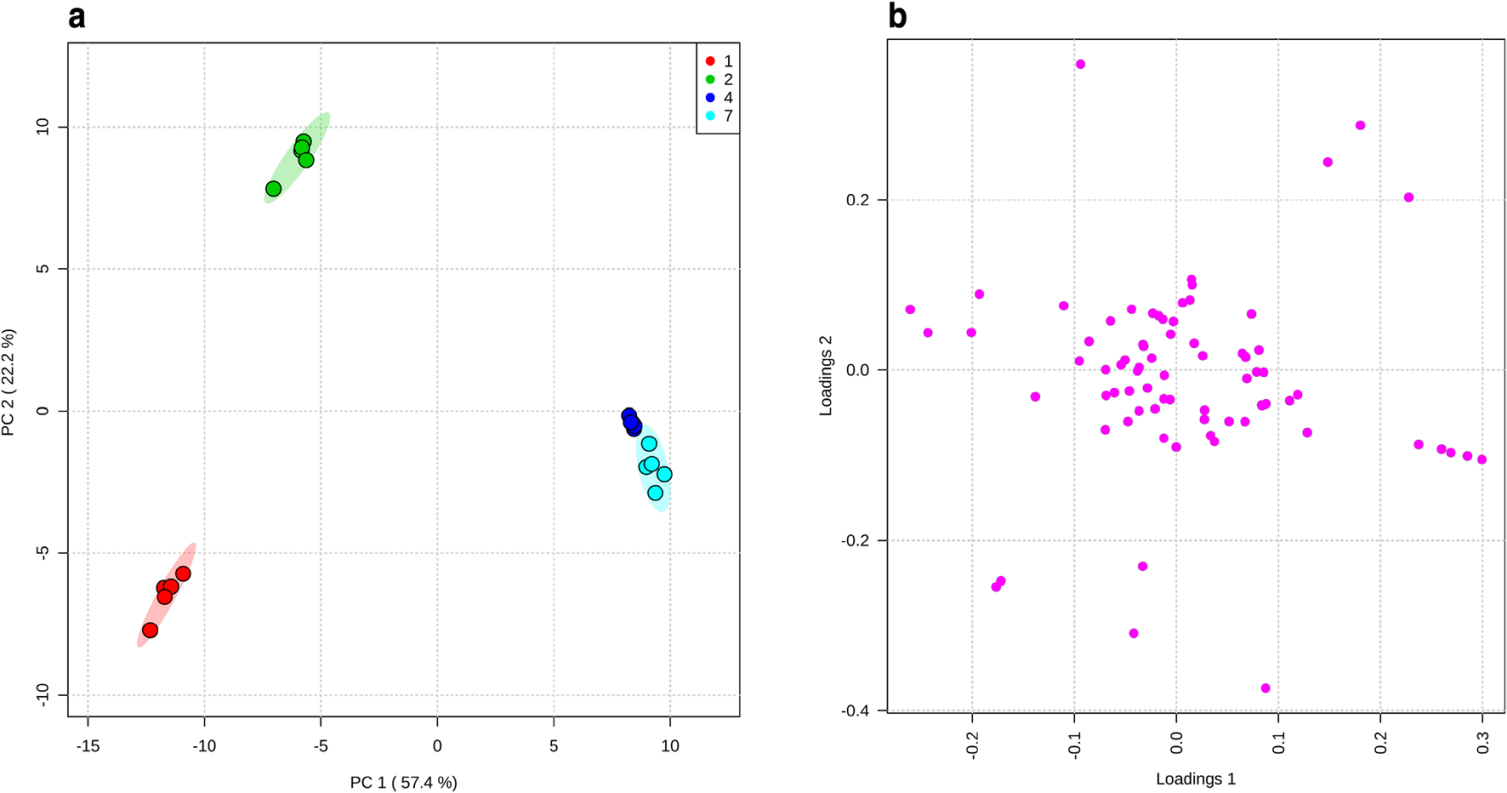
Principal component analysis. **a** Score plot showing the clustering pattern of *Xanthophyllomyces* wild-type strain at 1 (blue), 2 (yellow), 4 (green) and 7 (red) days of culture; **b** loading plot showing those metabolites responsible for the clustering observed in *Xanthophyllomyces*

In this study selected time points across the characteristic growth profile of *Xanthophyllomyces* have been used representing the lag (1 day) and exponential phases (2 days) as well as early (4 days) and late stationary (7 days) phases (Fig. S1). Visual inspection of the GS-MS chromatograms indicated similar profiles for all cultures (Fig. S2). A data matrix was created combining the variables from all the analytical platforms used and then the data subjected to PCA, in order to identify variables (metabolites) contributing to the overall changes in chemical composition between the different-growth stages (Fig. 4). The score plot from the PCA separate the cultures into three groups, 1, 2 and 4–7 days of cultivation. These data indicated that despite similar visual chromatographic profiles that the comparative chemical composition among all the cultures is significantly different. The loading plot of the PCA data indicated that the separation of the cultures could be attributed to several metabolites or sectors of metabolism. For example, glycerolipids, amino acid metabolites, sugars and some unknown compounds contributed to the clustering of the 1 day old culture from the other stages. While mainly free fatty acids, glycerolipids, ubiquinone, maltose, lysine and GABA contributed to the clustering of the 2 days old culture. The clustering of the 4 and 7 day old cultures was due to the carotenoids and members of fatty acid, organic acid, amino acid and unknown families.

More detailed analysis of the individual changes in metabolites was achieved through pair-wise statistical analysis, using the significance derived from Student’s *t* tests (Table 1). Glycerophospholipids, sugars, polyols, sugar alcohols, terpenoids and phosphorylated compounds decrease significantly with age of culture. Fatty acids increased in their quantity during the exponential phase and decreased during the stationary phase. While organic acids, sugar acids and non-amino acid N-containing compounds increase significantly 2.5-fold, threefold and fourfold, respectively. Terpenoids decreased in their total amount at day 2 and 4 of cultivation followed to a measurable increment from day 4 to day 7 of culture.

**Fig. 5:**
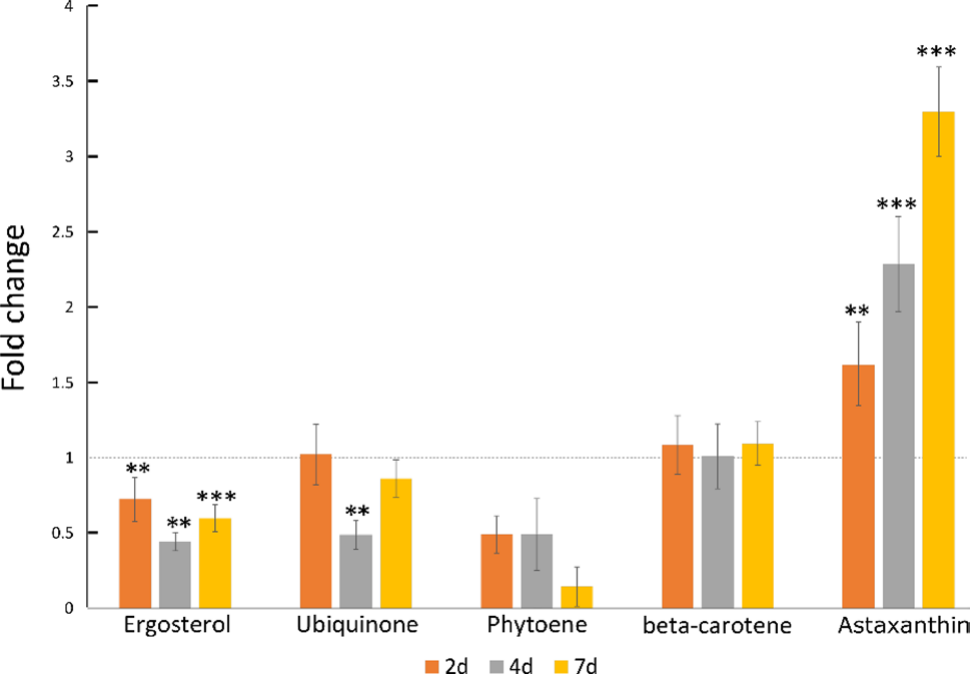
Specific changes in detectable terpenoids during growth. Data obtained by UPLC-PDA analysis. Fold changes of terpenoids using 1 day culture as control. *p\0.05; **p\0.01; ***p\0.001

More extensive analysis of the terpenoids (Fig. 5) indicated the strongly influence of ergosterol over the total amount of terpenoids produced. Ubiquinone exhibited lower amounts at the early stationary phase and reach the same level of lag and exponential phase at 7 days of culture. Astaxanthin is the main carotenoid in *Xanthophyllomyces* (Johnson 2003; Miller et al. 1976), phytoene and β-carotene are precursors which are identified in the method described. The amount of phytoene decreases over cultivation in comparison to day 1 of cultivation. β-Carotene is present and consistent at a similar levels throughout growth. Astaxanthin increased constantly over the 7 days of culture, constituting a 3.5-fold increase in the amount compared to day 1 of cultivation.

**Fig. 6:**
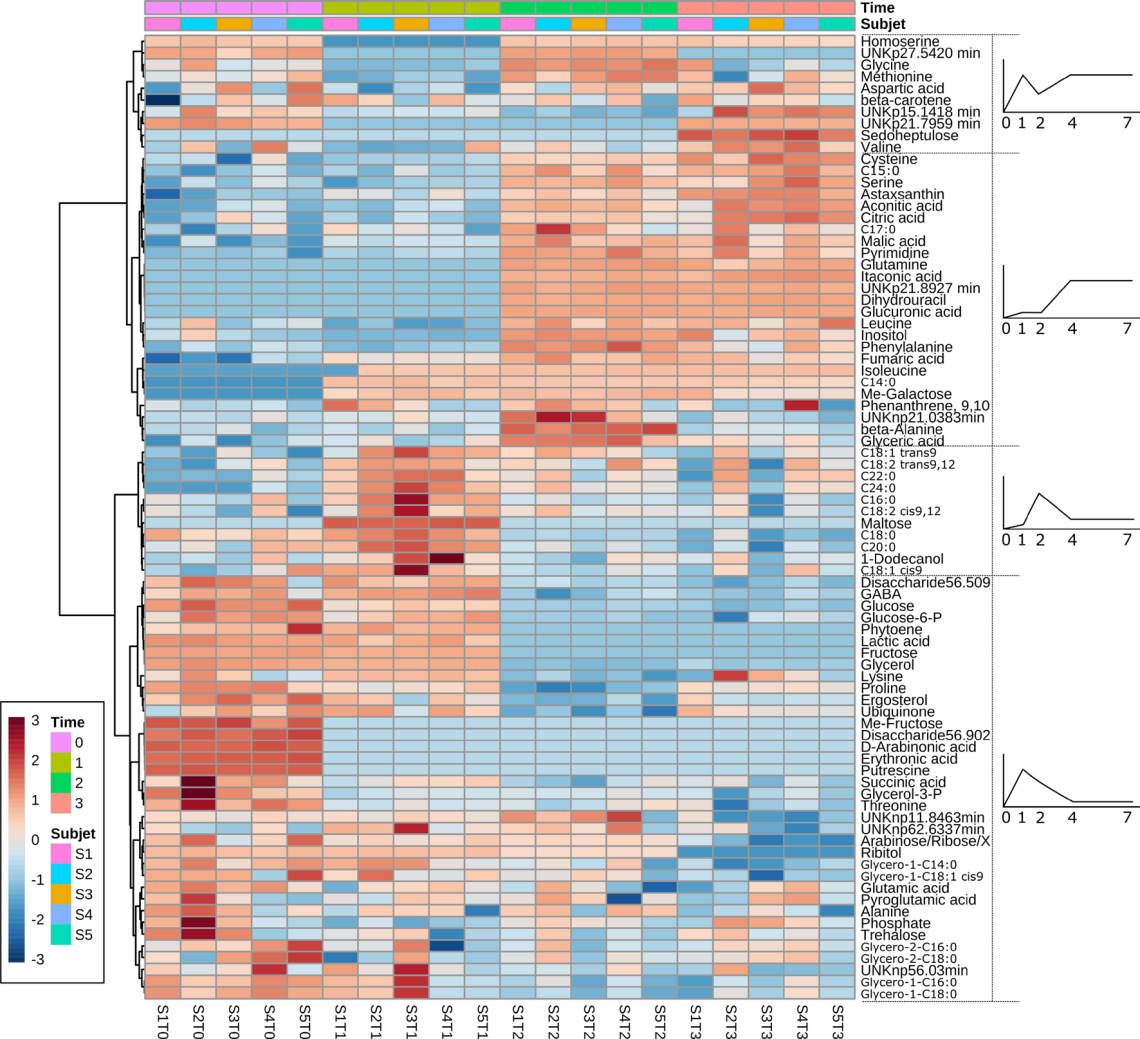
Heat map of the metabolite changes throughout the growth of *Xanthophyllomyces* in laboratory shake flask cultivation. Dashed line separate groups of metabolites with different trend (right) along of the culture; graphs represent the trend of the metabolites along the culture; numbers under the graph represent the day of the culture. The heat map was created using the server MetaboAnalyst and presented in Inkscape

These changes were then visualised by representing graphically the changes of metabolites and correlating with the growth curve of the cultures in a heat map (Fig. 6). The clustering of the samples distinguish two main metabolite groups based on the behaviour along the culture growth. One group correspond to those metabolites accumulated during the stationary phase: TCA, “short-chain” (14–17 carbon chain) saturated fatty acids, astaxanthin, β-carotene and most of the amino acids clustered in this group. The second cluster grouped metabolites synthetize rapidly during lag and exponential phase and metabolised during the stationary phase: “Long-chain” (more than 18 carbon chain) saturated fatty acids, glycerolipids, some amino acids, ergosterol and phytoene.

### Metabolic correlations associated with astaxanthin production

**Fig. 7:**
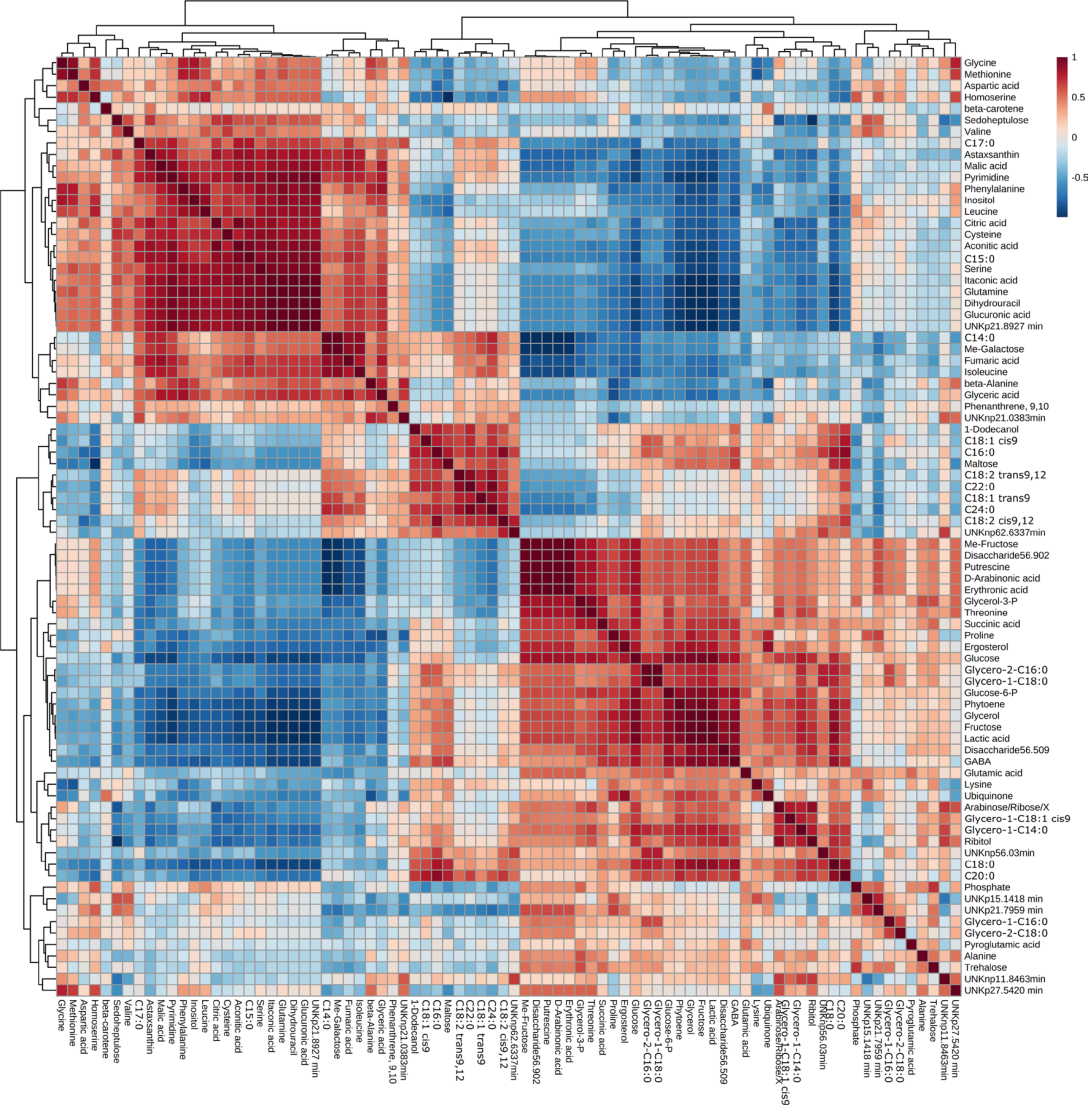
Heat map of correlation analysis of *Xanthophyllomyces* metabolites based on Pearson coefficients. The heat map was created using the server MetaboAnalyst and presented in Inkscape

Correlation analysis using Pearson coefficients was carried out and displayed in a heat map format (Fig. 7) associated analysis of compound patterns was made to correlate astaxanthin, with the rest of the metabolites (Table S3). Sugars, glycerolipids, “long-chain” saturated fatty acids, phytoene and ergosterol diminish in quantity as astaxanthin formation was favoured. Unsaturated and “long-chain” fatty acids, ubiquinone, some sugars and some amino acids did not show a significant positive or negative correlation with astaxanthin biosynthesis. The predominant metabolites/chemical classes observed over the cultivation period are summarised in Fig. S3. These data clearly displayed the predominant of metabolites present during the stationary phase.

## Discussion

In the present study a metabolite profiling workflow has been reported for the non-conventional industrial yeast *Xanthophyllomyces* which naturally produces astaxanthin, a high value carotenoid. Of the quenching methods tested washing with NaCl solution (W), followed by chloroform:methanol (CM) extraction were the most reliable method to arrest metabolites in *Xanthophyllomyces*, based on quenching recovery (0.84–0.94), extraction efficiency (1.00–1.41), number of targeted compounds (90) and chemical families representation. The need for specific quenching procedures for the microorganisms in question has been reported by (Mashego et al. 2007) and the present study corroborates these findings. Different methodology has been reported for metabolite profiling analysis in *Xanthophyllomyces* (Martinez-Moya et al. 2015). Pre-chilled methanol (60% v/v) for quenching the metabolism has been used previously (Martinez-Moya et al. 2015), similar to the quenching methods used in this study. In comparison to the evaluation of methods used for bacteria (Perez-Fons et al. 2014) all the quenching methods tested in the present study showed a very similar recovery parameters for the standards, suggesting that the metabolism of the yeast cell does not have the same rapidity and less susceptible to conditions adopted during harvesting. The quenching methods using methanol (M and M60), typically result in reduced metabolites, presumably because the approach, although rapid, can actually leach metabolites from the cell. Therefore, the simple washing of the cell with 0.9% (w/v) NaCl (W) to remove media components gave more representative results due to more efficient quenching of *Xanthophyllomyces* without perturbing metabolite levels.

It is important that the extraction method used provides a comprehensive representation of the main metabolite classes. Boiling ethanol was reported as an efficient extraction method in *Saccharomyces cerevisiae* (Gonzalez et al. 1997). In our study this method showed greatest variability in efficiency. This extraction method was also the least effective for *Mycobacteria* and *Bacillus* respectively (Drapal et al. 2014; Perez-Fons et al. 2014). Previous extraction methods applied to *Xanthophyllomyces* (Martinez-Moya et al. 2015) or to *Haematococcus pluvialis* (Lv et al. 2016) are based on sequential methanol:water extractions and focussed on polar metabolites. The implications associated with fatty acid components, glycerophospholipids, carotenoids and sterols in membranes has been reported (Gruszecki and Strzałka 2005), the presence of these compounds in the non-polar extraction validates the use of the present extraction method for non-polar components. Of the five different tested extraction methods showed altered affinities for different classes of metabolites. In *Xanthophyllomyces*, the dominating fatty acids are palmitic, oleic and linoleic acid (C16:0, C18:1, cis-9 and C18:2, cis-9,12, respectively) (Sharma et al. 2015), those compounds were clearly represented after CM extraction method. The efficiency of methanol:chloroform based method for the extraction of polar and non-polar metabolites has already been documented (Winder et al. 2008).

The construction of the AMDIS library is a valuable resource, enabling the identification of almost 70 metabolites from the chromatographic components. The optimised CM extraction method combined with UPLC, for terpenoids mainly, and GC–MS, for primary/intermediary metabolites, is a robust platform for studying the metabolism of *Xanthophyllomyces* and can hopefully be extended to other non-conventional industrially useful fungi.

In order to test the potential of the optimised system and define a baseline of steady state metabolite levels synthesised by *Xanthophyllomyces* under standardised laboratory cultivation conditions, the optimised CM method was used to analyse the biomass generated over the different growth phases (lag, exponential and early and late stationary phases) of the *Xanthophyllomyces* growth cycle.

From the collective PCA plots specific metabolite changes could be associated with different stages of growth. Direct pairwise comparisons of metabolites indicated the main metabolite changes contributing to the clustering were glycerophospholipids, sugars, polyols, sugar alcohols, terpenoids and phosphorylated compounds which decrease significantly with the time. Fatty acids increase in quantity during the exponential phase and decreased during the stationary phase. While organic acids, sugar acids and non-amino acid N-containing compounds increase significantly. Metabolite global changes were also reported previously in *Xanthophyllomyces* at different growth stages (Martinez-Moya et al. 2015).

Carotenoids are produced within the organism to provide protection against photooxidative stress and inactivated free radicals via electron transfer (Niyogi et al. 1997). The initiation of oxidative stress also stimulates the synthesis of carotenoids in fungi (Gessler et al. 2007). In *Xanthophyllomyces*, during late exponential phase carotenogenic-related and redox proteins have been reported to be more abundant than in previous growth stages (Martinez-Moya et al. 2015). Correlation analysis of metabolites to the astaxanthin (Fig. 7 and Table S3) lead to the separation of metabolites into two groups. Astaxanthin correlated positively with intermediary metabolism (TCA and amino acids). The accumulation of the TCA intermediates during the stationary phase may indicate a lower activity than in previous phases of cultivation. TCA and high respiratory cycles are related with ROS production, which increase the astaxanthin biosynthesis in *Xanthophyllomyces* (Schroeder and Johnson 1993). The close relation of carotenoid production, the TCA cycle and stress response has been reported with differential protein expression between wild-type and mutant strains (Barbachano-Torres et al. 2014).

In *Saccharomyces cerevisiae* proline protects the cell against different stress conditions such as oxidative or light induced stresses (Takagi 2008). In *Xanthophyllomyces*, proline diminished with the time of the culture (Table 1) correlating negatively with astaxanthin formation (Fig. 7 and Table S3), astaxanthin could provide more effective protection reducing the need for proline. The same effect has been described in the astaxanthin producing algae *Haematococcus pluvialis* (Lv et al. 2016).

Similar metabolite profiling techniques have been applied to a distant filamentous fungus *Phycomyces blakesleeanus* revealing global changes across intermediary metabolism of the mutants (Alcalde and Fraser 2016). Classical mutagenesis and genetic engineering have produced a plethora of strains with altered or enhanced carotenoid content in *Xanthophyllomyces* (Schmidt et al. 2011). A robust metabolite profiling method will enable a better understanding of the changes in steady state levels in *Xanthophyllomyces*.

A microbial-derived production platform for terpenoids could potentially replace plant-derived and chemically synthesised industrial methods (Ma et al. 2016). Nevertheless, the steady-state kinetic formation of terpenoids in microbial systems is still poorly understood (Zhu et al. 2014). Understanding these holistic changes will enable us to decipher the correlated metabolite pathways that exist and facilitate the design of new strategies for the exploitation of *Xanthophyllomyces* as a cell factory for high value isoprenoids. Moreover, important advances in proteomic (Martinez-Moya et al. 2011, 2015) and genomic (Bellora et al. 2016; Sharma et al. 2015) analysis in *Xanthophyllomyces* will greatly benefit from associated metabolite analysis in order to evaluate cellular regulation.

The growing interest in *Xanthophyllomyces* as a potential industrial producer of carotenoids and the importance of the “omics” technologies has been recently reported (Barredo et al. 2017). Metabolic engineering and new Synthetic Biology approaches can be applied to enhance specific pathways or to incorporate heterologous ones, which has already demonstrated in *Saccharomyces* (Meadows et al. 2016) and *E. coli* (Brunk et al. 2016; Ma et al. 2016; Zhu et al. 2014). The interaction between native biosynthetic pathways and those with the heterologous pathways incorporated is the mayor challenge for Synthetic Biology. The metabolite profiling method presented here provides an important component of the toolkit to approach the challenges of the microbial production of high value metabolites.

## Conclusion

This article reports procedures for the metabolite profiling of the non-conventional industrial yeast *Xanthophyllomyces dendrorhous*. These procedures have extended our ability to carry out holistic analysis across cellular regulation in this organism. The profiling procedure has been used to determine the relative changes in metabolites during it standardised laboratory growth conditions with defined lag, exponential and stationary phases. Interrogation of the data over this cultivation cycle has illustrated that the astaxanthin correlate positively with different sectors of intermediary metabolism (TCA and amino acids), “short-chain” saturated fatty acids and β-carotene, while other metabolites are diminished in quantity in favour of astaxanthin production; potential providing future targets for the manipulation of metabolism in this organism for the production of valuable compounds that are not directly associated with terpenoid biosynthesis.

## Experimental procedures

### Xanthophyllomyces strain and culture conditions

The wild-type *Xanthophyllomyces* strain CBS 6938 was used in this study. Starting from a pre-culture grown for 2 days and inoculation with a 1:10 ratio with the media. Cultivation was performed in baffled conical flasks (250 ml), containing YPD-medium (50 ml). Following inoculation cultures were incubated for 7 days at 22 °C in light (16–20 µmol m^−2^ s^−1^), while being shaken in an orbital manner at 140 rpm.

### The optimisation of a metabolite profiling procedure for xanthophyllomyces

**Fig. 8:**
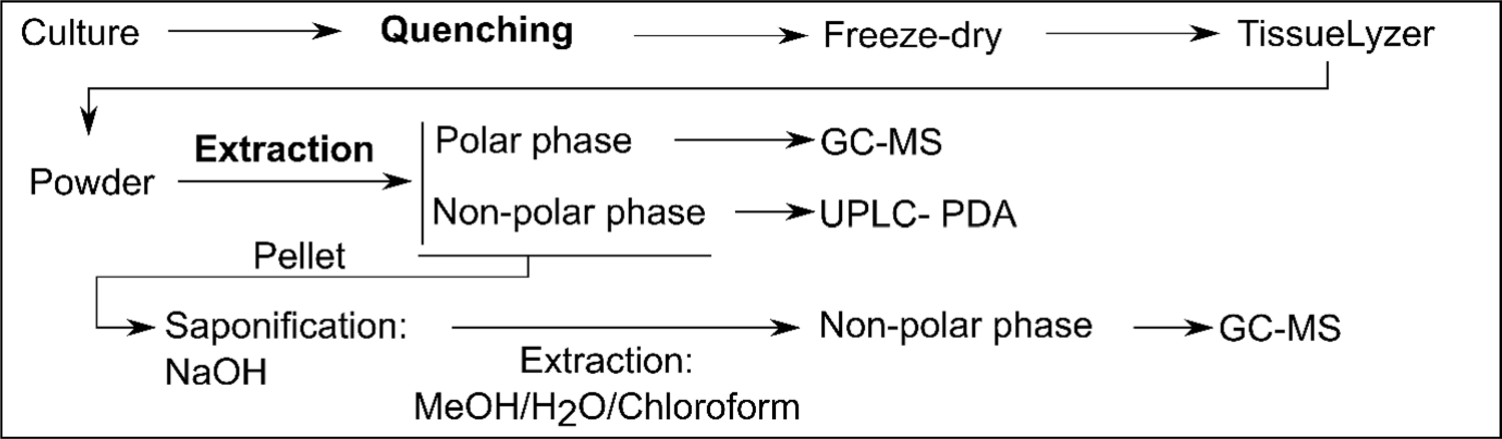
Quenching and extraction workflow scheme for *Xanthophyllomyces*

In order to evaluate procedures for the quenching of metabolites and then their efficient extraction, *Xanthophyllomyces* was cultivated in shake culture, harvesting the biomass during the stationary phase. To ensure accurate comparisons could be made the same biomass pool was used during quenching and extraction. Subsequently, cultivation in shake culture was standardised, ensuring designation of lag, exponential and stationary phases through the determination of OD measurements and colony forming units (CFU). Following robust reproducible sample preparation the analytical (GC–MS and UPLC) workflows and data analysis are summarised in (Fig. 8).

### Quenching the Xanthophyllomyces metabolome

Different quenching procedures were evaluated. Initially all procedures harvested *Xanthophyllomyces* biomass by centrifugation of the 50 ml cultures at 3500×*g* for 5 min at 4 °C (Eppendorf 5810 R, Hamburg, Germany). The remaining pellet was treated as follows: (i) a pre-chilled (− 20 °C) quenching solution, composed of 60% (v/v) methanol in 0.9% (w/v) NaCl totalling 30 ml was added. This approach was termed M60; while (ii) pre-chilled (− 20 °C) methanol (30 ml) was designated M; (iii) a wash solution (30 ml), comprised of 0.9% (w/v) NaCl, was termed (W). After centrifugation at 3500×*g* for 5 min at 0 °C, the supernatant was discarded and pelleted cells snap-frozen in liquid nitrogen, then freeze-dried and stored at − 80 °C until extraction. Non-treated snap-frozen pelleted cells were also used as a control (C).

The different quenching methods were evaluated by monitoring the recovery of four polar-metabolite standards, deuterated d_4_-succinic acid, d_2_-glucose, d_5_-glycine and ribitol, representing an organic acid, monosaccharide, amino acid and polyol chemistries, respectively. These classes of metabolites are rapidly affected by the metabolism. The procedure (Canelas et al. 2009) was used to quantify potential metabolite degradation and/or poor metabolic quenching.

Cultures were grown until stationary phase for optimal biomass. After quenching samples were freeze dried and chloroform:methanol extraction method (CM) was applied for polar extraction. The standards were added to the cells immediately after quenching and before analysis.

### Extraction of polar metabolites and carotenoids

The starting material for extraction was standardised, dried biomass (10 mg). The following approaches were tested:

(i) Chloroform:methanol (CM): 50% (v/v) methanol (500 µl) was added and placed in a sonication bath (Sonicor SC-120^TH^, Sonicor Instrument Corporation, Copiague, NY, US) at room temperature. Sonication was performed for 15 min at 50/60 Hz. Chloroform (1 ml) was added and the suspension centrifuged at 14,000 rpm for 5 min to facilitate phase separation. The epiphase (500 µl), containing polar metabolites, was separated from the organic phase (hyperphase) and stored at − 20 °C until further analysis. The hypophase was stored at − 20 °C and pooled with the non-polar extracts obtained as described in the following section.
(ii) Chloroform:methanol acidified (CMA): A modification of the chloroform:methanol method was tested by using 50% (v/v) methanol (500 µl) containing 0.1 M formic acid. The rest of the method proceeded as described for the CM method.
(iii) 80% (v/v) Methanol (80 M): 80% methanol (1 ml) was added and the suspension was sonicated for 15 min. The polar extract was collected after centrifugation (14,000 rpm, 5 min) and stored at − 20 °C prior to analysis.
(iv) Acidified acetonitrile:methanol:water (AAMW): A solution of acetonitrile:methanol:water (40:40:20) (v/v) containing 0.1 M formic acid (500 µl) was employed for extracting polar metabolites (Rabinowitz and Kimball 2007). Extracts were then sonicated (15 min) centrifuged (14,000 rpm, 5 min), the polar extracts removed and stored at − 20 °C prior to analysis.
(v) Boiling ethanol (BEt): 75% (v/v) ethanol (2 ml) was added. The suspension was heated at 95 °C for 3 min in a water bath and rapidly cooled on ice (Gonzalez et al. 1997; Taymaz-Nikerel et al. 2009). The ethanolic extract was then collected after centrifugation (14,000 rpm, 5 min) and taken to dryness. The sample was dissolved in distilled water (500 µl) and an aliquot (100 µl) used for analysis.

Cultures were grown until stationary phase for optimal biomass, quenching by washing with NaCl solution (W), performed and the material pelleted and then freeze-dried to eliminate any remain water and kept at − 80 °C before the extraction. The five different extraction methods based on different solvent solutions were tested. The same standards applied for the quenching were used. The standards were added to the cells immediately before extraction or prior to analysis (Fig. 1).

### Extraction of non-polar metabolites

After extracting polar metabolites the remaining pellets were saponified with aqueous 10% NaOH (w/v) at room temperature and sonicated for 10 min. The NaOH solution was removed by centrifugation and the pellet was extracted with chloroform:methanol (CM) as described in the previous section e.g. treatment (i). The hypophase, containing non-polar metabolites, was separated from the epiphase and stored at − 20 °C until further analysis. Phases for polar and non-polar metabolites obtained were dried under vacuum using GENEVAC EZ-2 Plus (GENEVAC LTD., Ipswich, England).

### Recovery and efficacy

Metabolite recoveries were calculated according to the following formula (Canelas et al. 2009):

$${\text{Recover}}{{\text{y}}_{{\text{x}};{\text{i}}}}={\left[ {\text{x}} \right]_{\text{i}}}{\text{AQ}}/{\left[ {\text{x}} \right]_{\text{i}}}{\text{BQ}}$$

where ‘‘x’’ indicates metabolite, ‘‘i’’ stands for the quenching method and the upper indexes BA and AQ meaning that the internal standards (IS) were added before analysis (BA) and after quenching (AQ), respectively. Recovery values much below 1 typically indicate degradation and/or poor quenching of metabolic activity. Similarly, the ability of different extraction methods to release metabolites from biological matrices can be evaluated in terms of efficacy as follows (Canelas et al. 2009):

$${\text{Efficac}}{{\text{y}}_{{\text{x}};{\text{i}}}}={\left[ {\text{x}} \right]_{\text{i}}}{\text{BE}}/{\left[ {\text{x}} \right]_{\text{i}}}{\text{BA}}$$

where ‘‘x’’ indicates metabolite, ‘‘i’’ stands for the extraction method and the upper indexes BA and BE meaning that the internal standards were added before analysis (BA) and before extraction (BE), respectively. Aliquots (100 µl) of BA samples were spiked with a solution (20 µl) of internal standards containing the deuterated standards, Deuterated d_4_-succinic acid, d_2_-glucose, d_5_-glycine and ribitol, at a concentration of 1 mg/ml each, giving a final amount of 20 µg of internal standard in the BA aliquots. The aliquots were then dried under vacuum and derivatised as described in next section. For BE and AQ series the solution of internal standards was prepared at 5 mg/ml and 20 µl used for spiking samples. Since solvent (500 µl) was employed for extraction the final concentration of IS was 0.2 µg/µl in the polar extract. An aliquot (100 µl) was evaporated to give a final amount of 20 µg of IS, that was derivatised concurrently with the rest of the components in the extracts as described below.

Dedicated experiments for calculating limit of detection and the limits of quantification were not performed concurrently in the present work as relative quantification (to internal standard) values were used routinely instead of absolute quantification using calibration curves for every metabolite detected. However, generic machine standard calibration curves show linearity in the range of 0.025–0.25 μg/μl for monosaccharides and 0.025–0.1 μg/μl for disaccharides, and an approximate LOD value can be extrapolated from these calibration curves to be in the range of 6–20 ppm.

### GC–MS analysis of polar and non-polar metabolites

Those polar and non-polar extracts, generated using the methods described in previous sections, were analysed by GC–MS as described in (Alcalde and Fraser 2016) with the following modifications. In brief an aliquot from each polar (200 µl) and non-polar (200 µl) extract were removed, dried under vacuum and solubilised in the derivatisation reagents. For example, samples were derivatised to their methoxylated and silylated forms according to (Halket et al. 2005). Methoxyamine hydrochloride (20 mg/ml in anhydrous pyridine; 30 µl) was added to samples and incubated at 40 °C for 1 h. Following this reaction, the suspensions were treated with MSTFA (70 µl) and heated at 40 °C for 2 h. The final solution (1 µl) was injected in split mode (1:10) into a 7890A GC on-line with a 5975C mass spectrometer (Agilent Technologies, Palo Alto, California, US). Metabolites were separated on a DB-5MS 30 m × 250 lm × 0.25 lm column (J&W Scientific, Folsom, California, US), equipped with a 10 m guard column and using a temperature gradient ranging from 70 to 320 °C at 5 °C min^−1^. Helium was employed as the carrier gas and the flow rate was 0.5 ml min^−1^. The inlet was heated to 280 °C and the mass spectrometer transfer line at 250 °C. A mixture of n-alkanes, ranging from 8 to 32 carbons, was used for retention index external calibration. Authentic standards, d_4_-succinic acid and d_27_-myristic acid for polar and non-polar extract, respectively, were added at a concentration of 1 mg/ml and aliquots (10 µl) to the samples before dried and subjected to the identical derivatisation procedure as that used with the extracts.

### Data analysis

Levels of metabolites analysed by GC–MS were quantified relative to the internal standard and corrected for the dried weight of the biomass. Compounds generating multiple peaks in the chromatogram as a consequence of the methoxylation and silylation reactions were quantified by summing the areas of the different derivatives. AMDIS (version 2.7) software was used for peak deconvolution and establishing the authors’ libraries for polar and non-polar metabolites. Identification of metabolites for library construction was done by comparing mass spectra and retention indexes to NIST [version 2.0 (2008)] and Golm Metabolome (http://gmd.mpimp-golm.mpg.de/analysisinput.aspx) mass spectral databases and confirmed with authentic standards whenever possible. Those compounds not identified were named as UNK followed the sub index p or np for polar or non-polar extracts, respectively, and by the corresponding retention time. The identification criteria used and annotation of unknowns was performed as described by Bino et al. (2004) and Sumner et al. (2007). Data matrices were transformed using the pareto-scaled method (van den Berg et al. 2006) and log transformation (Fig. S4), multivariate analysis, time course heat map and correlation analysis performed using the server MetaboAnalyst 3.0 (http://www.metaboanalyst.ca) (Xia et al. 2016). Means, standard deviation, p values and q values (for FDRs, Benjamini and Hochberg 1995) were calculated in Excel. Extraction-method heat map was created with Excel and Inkscape.

### Separation and detection by UPLC-PDA

Carotenoids, ergosterol and ubiquinone (Coenzyme Q9) extracted by the method described previously, were separated and identified by Liquid Chromatography with photodiode array detection. An Acquity ultra high performance liquid chromatography system (Waters) was used with an Ethylene Bridged Hybrid (BEH C18) column (2.1 × 100 mm, 1.7 mm) with a BEH C18 VanGuard precolumn (2.1 × 50 mm, 1.7 mm). The mobile phase used was A, methanol/water (50/50), and B, acetonitrile (ACN)/ethyl acetate (75:25) and the flow rate was 0.5 ml min^−1^. All solvents used were HPLC grade and filtered prior to use through a 0.2-mm filter. The gradient was 30% A:70% B for 0.5 min and then stepped to 0.1% A:99.9% B for 5.5 min and then to 30% A:70% B for the last 2 min. Column temperature was maintained at 30 °C and the temperature of samples at 8 °C. Online scanning across the UV/visible range was performed in a continuous manner from 250 to 600 nm, using an extended wavelength photo diode array detector (Waters, Watford, UK).

## Electronic Supplementary Material

Below is the link to the electronic supplementary material.

Supplementary material 1 (XLSX 43 KB)

Supplementary material 2 (XLSX 38 KB)

Supplementary material 3 (XLSX 14 KB)

Supplementary material 4 (DOCX 1621 KB)

## Abbreviations

GC/MS: Gas chromatography mass spectrometry
UPLC: Ultra performance liquid chromatography
OD: Optical density
TCA: Tricarboxylic acid
