# Supplementary material for: Extending our tools and resources in the non-conventional industrial yeast Xanthophyllomyces dendrorhous through the application of metabolite profiling methodologies
Source: Metabolomics. 2018 Feb 12;14(3):30. doi: 10.1007/s11306-017-1313-9 (PMC5809543; doi:10.1007/s11306-017-1313-9)
Supplement: Supplementary file 4 — Supplementary material 4 (DOCX 1621 KB) [file 11306_2017_1313_MOESM4_ESM.docx]

Title: Extending our tools and resources in the non-conventional industrial yeast *Xanthophyllomyces dendrorhous* through the application of metabolite profiling methodologies.

Eugenio Alcalde and Paul D. Frase


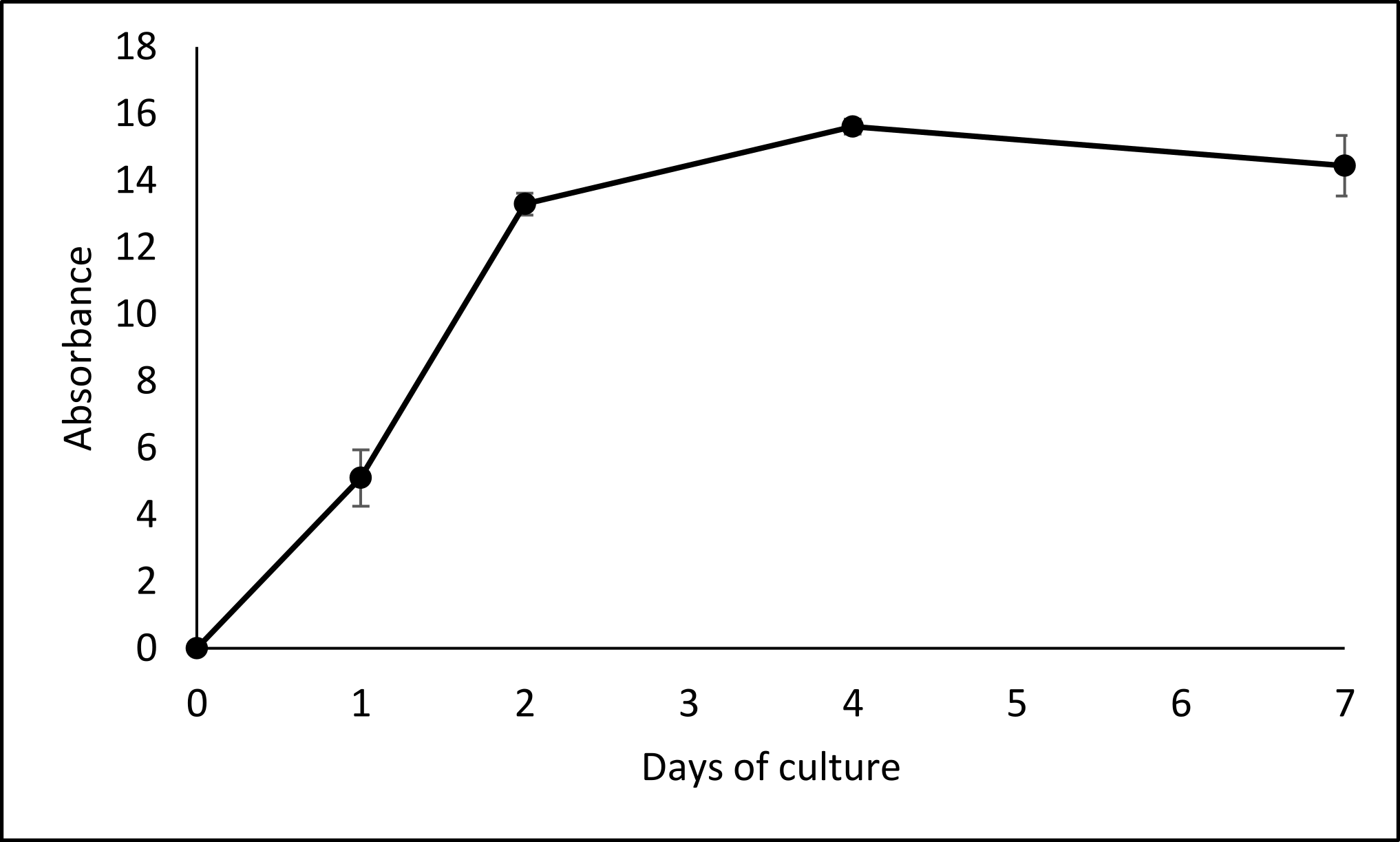


**Supplementary Figure 1. Culture growth during laboratory shake flask cultivation.** Optical density (600 nm) at one, two, four and seven day culture. Mean and standard deviation of three biological replicates.





**Supplementary Figure 2.** Gas Chromatography fractionation of the polar and non-phase of the *Xanthophyllomyces* one, two, four and seven days cultures.


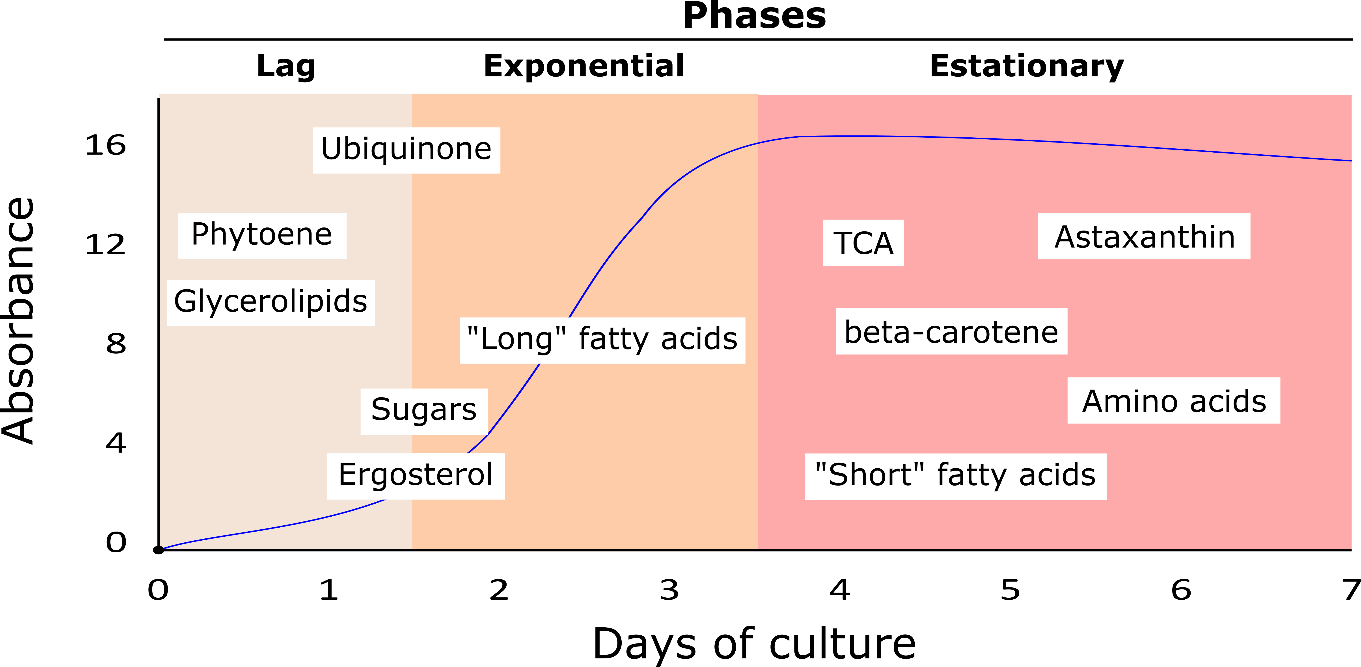


**Supplementary Figure 3 – Distribution of metabolites along the growth of *Xanthophyllomyces***


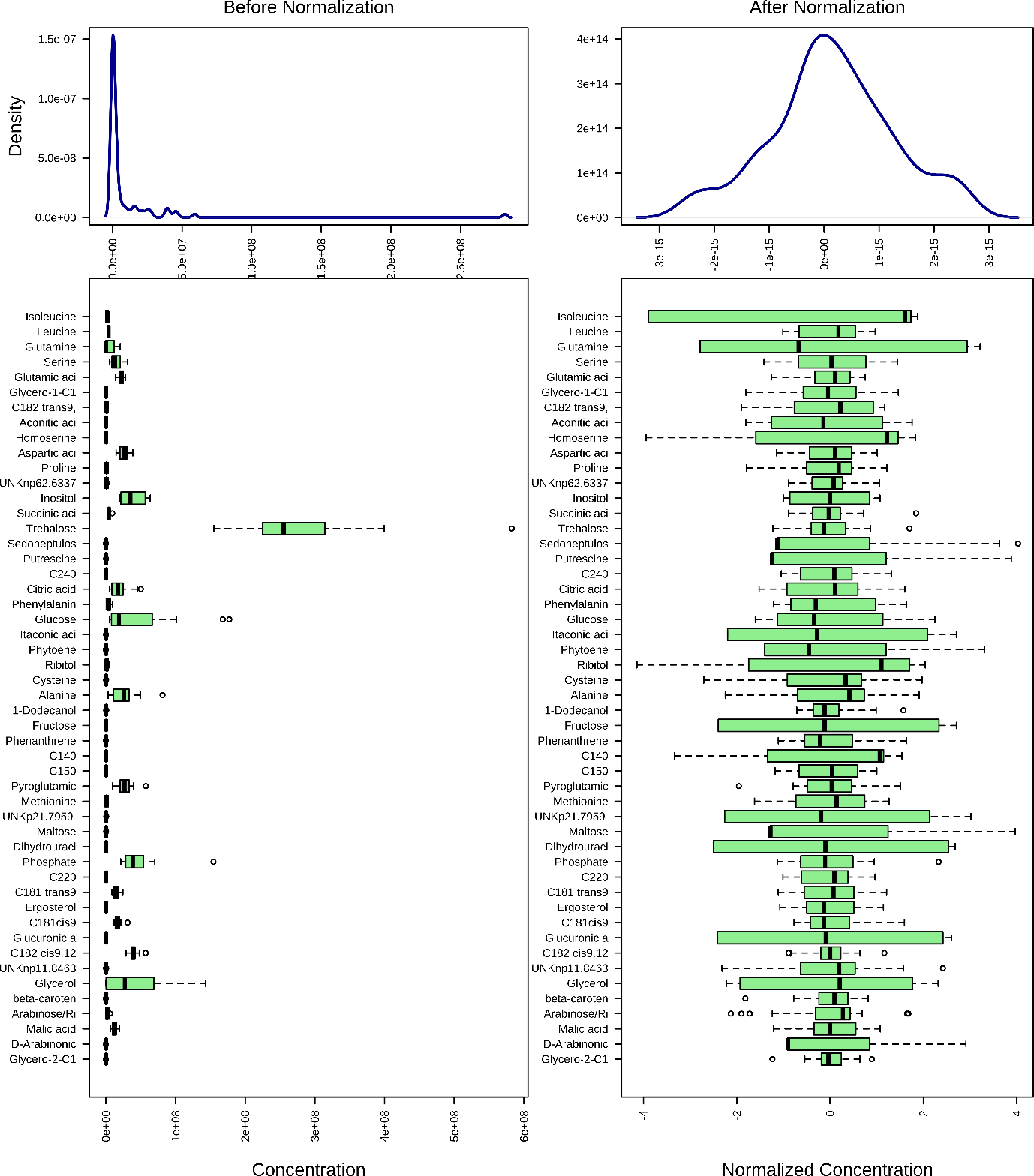


**Supplementary Figure 4. Data transformation and normalization.** Example of 60 metabolites before and after the normalization by log transformation and pareto-scaled method (van den Berg et al. 2006) using the server MetaboAnalyst.
